# Endogenous n-3 polyunsaturated fatty acids protect against imiquimod-induced psoriasis-like inflammation via the IL-17/IL-23 axis

**DOI:** 10.3892/mmr.2014.2136

**Published:** 2014-04-09

**Authors:** SI QIN, JU WEN, XIAO-CHUN BAI, TIAN-YU CHEN, RONG-CHANG ZHENG, GUI-BIN ZHOU, JING MA, JIE-YING FENG, BI-LING ZHONG, YI-MING LI

**Affiliations:** 1 Department of Dermatology, Southern Medical University Affiliated Guangdong Provincial No. 2 People’s Hospital, Guangzhou, Guangdong, P.R. China; 2 The Third College of Clinical Medicine, Southern Medical University, Guangzhou, Guangdong, P.R. China; 3 Department of Cell Biology, School of Basic Medical Science, Southern Medical University, Guangzhou, Guangdong, P.R. China; 4 Department of Orthopaedics, The Third Affiliated Hospital of Southern Medical University, Guangzhou, Guangdong, P.R. China; 5 Department of Pathology, Guangdong Provincial No. 2 People’s Hospital, Guangzhou, Guangdong, P.R. China

**Keywords:** fat-1 mice, inflammation, n-3 fatty acids, psoriasis, Th17 cells

## Abstract

The beneficial effects of n-3 polyunsaturated fatty acids (PUFAs) on psoriasis have been reported in rats, mice and humans, but the specific mechanisms involved have not been well defined. The present study utilized the fat-1 mouse, a transgenic model that can endogenously convert n-6 FAs into n-3 PUFAs, to directly determine if the outcomes of psoriasis were correlated with n-3 PUFAs. Wild-type (WT) and fat-1 mice, which were treated daily with imiquimod (IMQ) cream or control cream on the shaved right ear and dorsal skin, were fed the same diet. The severity of inflammation of the ear and dorsal skin was scored according to the clinical Psoriasis Area and Severity Index (PASI) and epidermal hyperplasia was measured by H&E staining. The expression of inflammatory factors in the epidermis was analyzed by immunohistochemical analysis. Flow cytometry and an enzyme-linked immunosorbent assay were used to measure the differences in the content of inflammatory factors in the blood serum and to determine which of CD4^+^ T cells were present in the spleen between IMQ-induced fat-1 mice and WT mice. Fat-1 IMQ-induced mice exhibited significantly lower levels of inflammatory cell-like T helper 17 cells (Th17 cells) and higher levels of regulatory T cells (Treg cells) in the spleen as compared with the WT IMQ-induced mice. n-3 fatty acids stimulated Th17 cells to produce lower levels of inflammatory factors, including interleukin (IL)-17, IL-22, IL-23 and stimulated Treg cells to produce higher anti-inflammatory factors, such as Foxp3. In conclusion, the present study provides further insight into the mechanisms involved in preventing inflammation in psoriasis-like mice by n-3 PUFAs using a fat-1 transgenic mouse model.

## Introduction

Psoriasis, which is regarded as a T-cell-mediated inflammatory skin disease, is characterized by hyperproliferation and poor differentiation of epidermal keratinocytes, affecting ≤2% of the population in Northern European countries. It is defined as an immunological disease, that is coupled with prominently increased vascularization of the skin, fibroblast activation and leucocyte infiltration. The underlying pathogenic mechanisms of this condition have not, however, been entirely clarified. Recently, numerous studies have confirmed that T helper 17 (Th17) cells and the inflammatory factors it produces, including interleukin (IL)-17, IL-22 and IL-23, are detected in psoriatic skin lesions, serum and are implicated in psoriasis pathogenesis (1–8).

Currently, it is well established that omega-3 long-chain polyunsaturated fatty acids (PUFAs) have a potential role in the treatment of numerous diseases, including non-alcoholic fatty liver disease (9), chronic experimental colitis (10), diabetes (11) and pancreatic ductal adenocarcinoma (12). n-3 PUFAs exert their beneficial effects by inhibiting the actions of numerous different cytokines in disease progression and are also essential fatty acids to normal physiological functioning. n-3 PUFAs include alpha-linolenic acid (ALA), eicosapentaenoic acid (EPA) and docosahexaenoic acid (DHA). Evidence suggests that n-3 PUFAs are promising candidates as a safe adjuvant holistic therapy for psoriasis, either as an active anti-inflammatory agent by itself or as a dual action synergistical enhancer for other anti-psoriatic treatments. The roles of n-3 PUFAs are highly diverse, including the maturation and differentiation of the stratum corneum, the inhibition of proinflammatory eicosanoids and cytokines, and the maintenance of the stratum corneum permeability barrier (13). Mammals are not able to endogenously synthesize n-3 PUFAs nor convert n-6 FA to n-3 PUFAs, on account of lacking n-3 desaturase, the enzyme that catalyzes this reaction. Therefore, the majority of the studies with n-3 PUFAs in psoriasis utilize oral, intravenous and topical preparations. In the majoriy of these investigations, n-3 PUFAs are associated with an observed improvement in patient mean Psoriasis Area and Severity Index (PASI) score, as well as in clinical symptoms, including pruritus. Despite the majority of studies adopting more mature models, the formulation associated with the fat content isocaloric diet is confounded. Therefore, it is difficult to control the fat composition between the control and experimental diets.

In 2004, Kang *et al* generated transgenic fat-1 mice based on C57BL6 mice, carrying the fat-1 gene, which encodes for an n-3 desaturase from *Caenorhabditis elegans* (14). Fat-1 transgenic mice have an n-6/n-3 fatty acid ratio of 1:1 compared with wild-type mice with a ratio of 20–30:1. In fat-1 transgenic mice, n-3 fatty acids are endogenously synthesized, which leads to an increase in n-3 PUFAs and a decrease in n-6 fatty acids, and subsequently, a reduction in the n-6/n-3 fatty acid ratio. As a result, the fat-1 mouse model may avoid the potential confounding factors associated with other models, including diet, because the same diet is provided to the wild type (WT) and fat-1 mice. Therefore, the fat-1 mouse represents a significant advance in the development of a more sophisticated model to investigate the effect of n-3 PUFAs and n-6/n-3 FA ratios on physiological parameters, including molecular mechanisms, without the necessity of providing exogenous n-3 fatty acids. Despite promising accumulating evidence on the potential benefits of n-3 PUFAs in psoriasis, the underlying mechanisms of this effect remain elusive. In the present study, we used this fat-1 transgenic psoriasis mouse model to establish n-3 PUFAs as a therapeutic agent for psoriasis and to examine the molecular mechanisms underlying this effect.

## Materials and methods

### Animals and treatments

**Figure 1:**
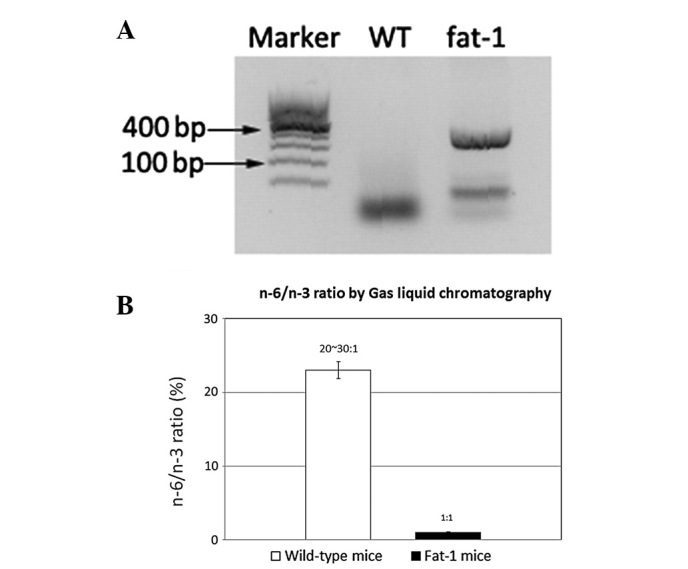
(A) Identification of the fat-1 gene by PCR; screen of fat-1 gene positive C57BL6 mice and fat-1 gene negative C57BL6 mice. (B) Gas liquid chromatography measured the amount of n-3 PUFAs and n-6 PUFAs of the mouse tail. PUFAs, polyunsaturated fatty acids; PCR, polymerase chain reaction.

Fat-1 transgenic mice and C57BL6 WT control mice were obtained from Professor Yifan Dai (15) and bred in the Southern Medical University’s laboratory animal facility (Guangdong, China). Male fat-1 transgenic mice were mated with wild-type C57BL6 female mice to obtain female fat-1 positive C57BL6 mice (fat-1) and fat-1 negative C57BL6 mice (WT) identified by genotyping using a polymerase chain reaction (PCR) kit purchased from (Takara Bio, Inc.; Dalian, Liaoning, China; Fig. 1). The fatty acid composition of the mouse tails was measured utilizing gas chromatography (GC; Fig. 1) (16). Weight-matched mice were housed in a laboratory animal care facility in cages (n=4/cage), in pathogen-free conditions and subject to a 12 h light/dark cycle at 24°C and provided with food and water *ad libitum*. At the age of 8 weeks, 48 mice (24 WT mice and 24 fat-1 mice) received a daily topical dose of 62.5 mg of commercially available imiquimod (IMQ) cream (5%; Aldara; 3M Pharmaceuticals, St. Paul, MN, USA) or control cream (Vaseline cream; Unilever, Greenwich, CT, USA) on the shaved right ear and dorsal skin for 14 consecutive days. This dose (translating in a daily dose of 3.125 mg of active ingredients) was empirically ascertained to induce optimal and reproducible skin inflammation in the mice (17). They were divided into the following four groups (n=12/group): Group A, WT cream; Group B, WT IMQ; Group C, fat-1 cream and Group D, fat-1 IMQ.

A total of 48 mice were separately fed with a general diet for two weeks prior to being sacrificed. The present study was approved by the Animal Research Ethics Committee of Southern Medical University (Guangdong, China) and the principles of the National Institutes of Health Guide were strictly followed in all experimental procedures.

### Scoring severity of skin inflammation

The clinical PASI is a useful tool as an objective scoring system for the severity of inflammation of the dorsal and ear skin in mice. The fixed area with IMQ treatment was accounted for in the overall score, excluding the affected skin area. Erythema, scaling and thickening were calculated respectively on a scale from 0–4: 0, none; 1, slight; 2, moderate; 3, marked and 4, highly marked. The cumulative scores (the amount of erythema, scaling and thickening) acted as a measure of the severity of inflammation. Repeated measurements of the thickness of the right ear with a micrometer (Mahr) were conducted for comparison with the left ear.

### Enzyme-linked immunosorbent assay

Blood serum was obtained on the day of sacrifice by means of retroorbital bleeding from anesthetized (isoflurane) mice. Serum was obtained by centrifugation at 300 × g for 15 min at 4°C, then evaluated for the levels of inflammatory factors. Serum IL-17, IL-22 and IL-23 were measured using the Quantikine Mouse IL-17A/F kit (M17AF0; R&D Systems, Collegeville, PA, USA), Quantikine Mouse IL-22 kit (M2200; R&D Systems) and Quantikine Mouse IL-23 kit (M2300; R&D Systems).

### Measurement of body weight and spleen weight

At the beginning of the treatment, the WT and fat-1 mice were weight matched using a CS 200 balance (Ohaus, Pine Brook, NJ, USA). The spleens were carefully obtained and weighed at the time of sacrifice using a Mettler balance (Mettler Toledo, Columbus, OH, USA).

### Flow cytometry

Spleen samples were ground with 200 m meshs and a syringe piston to obtain single-cell suspensions. Cells were stimulated with 50 ng/ml of PMA (Sigma, St. Louis, MO, USA) and 1 μg/ml of ionomycin (Sigma) in the presence of Monensin (BD Golgistop™ protein transport inhibitor), in complete RPMI-1640. The cells were then diluted to 10 million cells/ml, and centrifuged at 300 × g for 5 min and the buffer was removed. The cell pellet was carefully suspended in the residual volume of staining buffer and then 200 μl of freshly prepared cold 1X BD Pharmingen™ Mouse Foxp3 fixation buffer was added. To fix the cells, they were then incubated for 30 min at 4°C in the dark. Then, the sample was centrifuged at 300 × g for 5 min and the fixative was removed. To permeabilize the cells, careful suspension of the cell pellet in another 200 μl of freshly prepared pre-warmed (37°C) 1X BD Pharmingen™ Mouse Foxp3 permeabilization buffer was repeated and the cells were then incubated for 30 min at 37°C in the dark. Next, the cells were centrifuged at 300 × g for 5 min and the buffer was removed. To wash the cells, 200 μl of BD Pharmingen™ stain buffer (FBS) was added to each tube, centrifuged at 300 × g for 5 min and then the buffer was removed. A total of 20 μl/test of the mouse Th17/Treg phenotyping cocktail or the appropriate negative staining control was added and the cells were incubated at room temperature (RT) for 30 minutes in the dark. Cells were protected from light throughout the staining and storage, and the washing of the cells was repeated twice. The cell pellet was suspended in 200 μl stain buffer and was proceeded by flow cytometry (BD) and analysis with CellQuest software (BD). The viability of the cells was examined by staining with propidium iodide.

### Histology and immunohistochemistry

The dorsal and ear tissues were formalin-fixed and paraffin-embedded, and stained with H&E. Epidermal thickness was accurately measured by ImagePro Plus software (Leeds Precision Instruments, Minneapolis, MN, USA). The total epidermal area was calculated using a series of rectangles and the data was divided by the total length of the epidermis.

For immunohistochemistry, sections from the ear and dorsal tissues were deparaffinized with xylene and rehydrated, and then hydrated with a graded alcohol series. The ear and dorsal sections were incubated in 10 ml citric acid (pH 6.0) at 95°C for 30 min to unmask antigens and the endogenous peroxidase activity was quenched by treating sections with 3% hydrogen peroxide at RT for 5 min. The sections were blocked at RT for 60 min followed by incubation with primary antibodies (Abs): rabbit anti-mouse IL-17A polyclonal Ab (H-132; Santa Cruz Biotechnology, Inc., Santa Cruz, CA, USA), rabbit anti-mouse IL-22 polyclonal Ab (ab18564; Abcam, Cambridge, MA, USA) and rabbit anti-mouse IL-23 polyclonal Ab (H-113; Santa Cruz Biotechnology, Inc.). This was followed by treatment with horseradish peroxidase-linked secondary anti-rabbit GT Vision™ II polymer (Dako, Carpinteria, CA, USA) and DAB substrate kit for peroxidase (Dako).

### Statistical analysis

Results are expressed as the mean ± SEM and data analysis was performed with the SPSS 13.0 software (SPSS, Inc., Chicago, IL, USA) and ANOVA. P<0.05 was considered to indicate a statistically significant difference.

## Results

### Effect of endogenous n-3 desaturase on n-6/n-3 ratios in mice

From the PCR analysis (Fig. 1A), it was possible to screen the fat-1 gene positive C57BL6 mice and fat-1 gene negative C57BL6 mice. Gas liquid chromatography measured the quantity of n-3 PUFAs and n-6 FAs of the mice tails. The n-3 PUFAs and n-6 PUFA ratios in WT mice was ~20–30:1, whereas in the fat-1 mouse this ratio was 1:1 (Fig. 1B). The amount of n-3 PUFAs was enhanced, in contrast with the levels of n-6 FA, which were decreased, which is a result that may be attributed to the presence of n-3 desaturase in the transgenic mice model. A total of 48 mice were grouped utilizing this method in order to obtain accurate results.

### Effect of endogenous n-3 PUFAs on the structural features of IMQ-induced skin inflammation in mice

**Figure 2:**
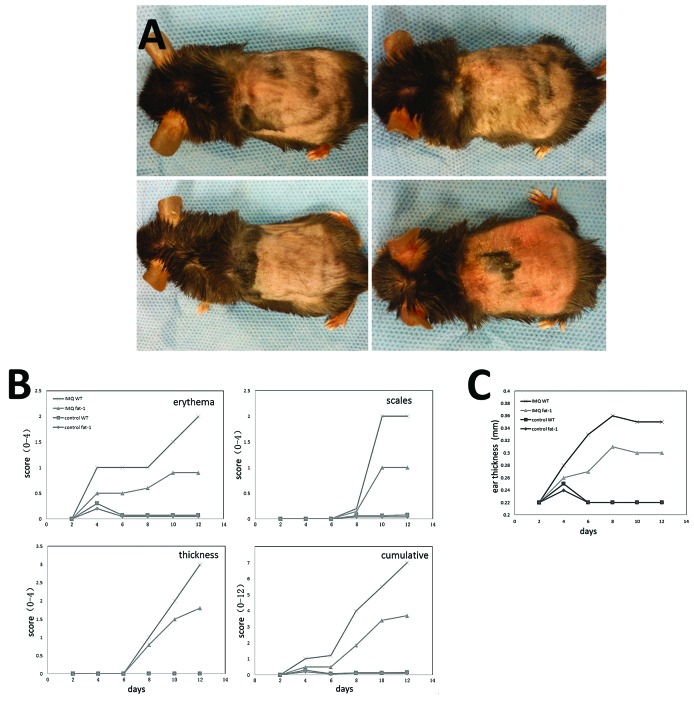
Skin inflammation in C57BL6 mice caused by IMQ phenotypically resembles psoriasis lesions in humans. Fat-1 and WT mice were treated daily with IMQ cream or Vaseline (control cream) on the shaved right ear and dorsal skin. (A) Representative macroscopic views of mouse dorsal skin following continuous treatment for 14 days. (B) Epidermal thickness and erythema, scaling of the dorsal skin were respectively identified on the days indicated on a scale from 0–4. The cumulative score (the three mentioned above) is represented. (C) Thickness of the IMQ-treated ear was measured daily. IMQ, imiquimod; WT, wild-type.

In the present study, IMQ cream and Vaseline cream were applied on the shaved right ear and dorsal skin of WT mice and fat-1 mice for 14 consecutive days. Three days following the application of IMQ onto the ears and dorsal skin of the mice, these areas began to exhibit symptoms of erythema, scales and thickening. These signs of inflammation, as observed in groups B and D mice phenotypically resemble psoriasis. Mice in group D were notably milder than in group B, who resembled the mice from groups A and C, where their ears and dorsal skin remained smooth (Fig. 2A). From the images revealed in Fig. 2B, it is possible to detect and quantify the severity scores of the mice. Signs of inflammation in groups B and D continually increased in severity until the end of the study. Mice in group B had higher scores than group D, while the mice in the control groups (A and C) treated daily with Vaseline cream did not present with any signs of inflammation. Additionally, compared with group A and C, the thickness of the right ear of mice who received daily IMQ-treatment increased from days 5–6 onward and were recorded, which in group B was more notable (Fig. 2C).

As van der Fits *et al* (17) described in 2009, IMQ-induced cutitis in mice resembled psoriatic lesions in human patients, not only in respect of the phenotypic symptoms, but also the histological characteristics. Furthermore, the development of the lesion was closely associated with the levels of IL-23 and IL-17 (18). In the present study, utilizing this model produced consistent results, because the inflammation of mice in group D was milder than in group B. The only difference between the two types of mice was the n-6/n-3 PUFA ratios, which resulted in differential responses to the daily IMQ-treatment. The data revealed endogenous n-3 PUFAs may protect against psoriasis-like lesions by means of its anti-inflammatory action.

### Effect of endogenous n-3 PUFAs on increased inflammatory cytokines in serum

**Figure 3:**
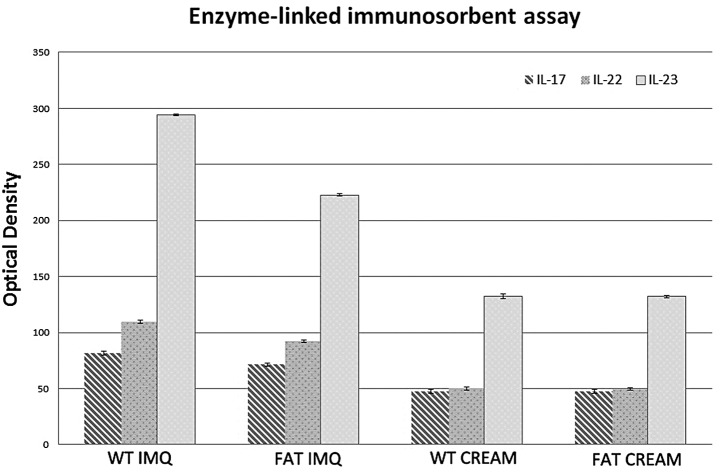
Mice were sacrificed and the levels of inflammatory cytokines in the serum were determined. Four groups of mice were treated by IMQ or Vaseline for 14 days. The bar graph demonstrates that IL-17, IL-22 and IL-23 in serum of mice in different groups have different conditions. IMQ, imiquimod; WT, wild-type; IL, interleukin.

The levels of IL-17, IL-22 and IL-23 in IMQ-treated groups were significantly higher (P<0.05) in the serum than in the control groups (Fig. 3). The levels of the inflammatory factor associated with Th17 cell in the serum of fat-1 IMQ-treated mice were significantly lower (P<0.05) compared with that in WT IMQ-treated mice. There was no significant difference (P<0.05) between WT and fat-1 control mice in the serum inflammatory factor levels. IL-17, IL-22 and IL-23 secreted by Th17 cells in the serum of mice in group A and C remained at low levels prior to their mortality. It is possible that endogenous n-3 PUFAs prevent Th17 cells from producing inflammatory factors, such as IL-17. By contrast, endogenous n-3 PUFAs may perform a particular role in decreasing the differentiation of CD4^+^ cells into Th17 cells.

### Effect of endogenous n-3 PUFAs on spleen weight in an IMQ-induced psoriasis model

**Figure 4:**
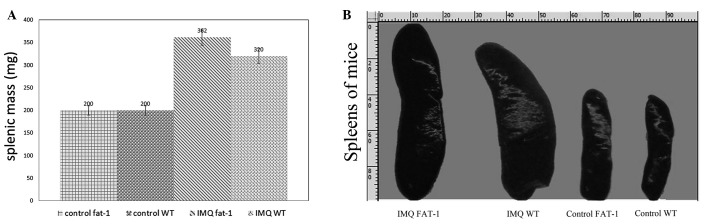
Changes in the mass and size of the spleen induced by IMQ treatment. The four groups of mice were treated with IMQ or Vaseline for 14 consecutive days. (A) Spleen mass was recorded when the mice were sacrificed. (B) Size of spleens with comparison in image direct-viewing. IMQ, imiquimod; WT, wild-type.

The mice in the four groups were subject to topical IMQ treatment and any significant changes either in the size or the weight of spleens were observed. At first, from direct-viewing of the images, it is possible to note that the lengths of spleens of mice in group B and D were all ≥22 mm, while the spleens in group A and C measured ≤16 mm, which was approximately two thirds of the IMQ-treatment mice (Fig. 4A). There was a significant difference in the value for spleen weight between the fat-1 and WT IMQ-treatment mice (P<0.05) following 14 days of treatment, however, there is no significant difference in the spleen weight between fat-1 and WT cream-treatment mice (P>0.05). IMQ-treatment groups induced an increase in the spleen weight compared with the control groups (Fig. 4B).

The spleen is the largest immune organ in the human body, owing to various immunocompetent cytokines, which have an important role in anti-infection and anti-tumor activities. The increased weight and size observed in the IMQ-treated mice, suggests that the amount of cells in spleen had increased to a high level, which may be a sign of enhancing the immunoreaction.

### Effect of endogenous n-3 PUFAs on IMQ-induced splenomegaly with increased numbers of Th17 cells

Following 14 days of IMQ and Vaseline treatment, we identified a consistently significant spleen enlargement in WT mice and fat-1 mice (Fig. 4A). To determine the percentages of CD4^+^T, Treg and Th17 cytokine positive cells in the spleen, splenic cells were activated *ex vivo* by phorbol myristate acetate (PMA; protein kinase C activator) plus ionomycin (Ca^2+^ ionophore), stained intracellularly for ‘Mouse Th17/Treg Phenotyping Cocktail’ containing ‘Mouse CD4 PerCP-Cy5.5’, ‘Mouse IL-17A PE’ and ‘Foxp3 Alexa Fluor^®^ 647’ and analyzed using flow cytometry.

**Figure 5:**
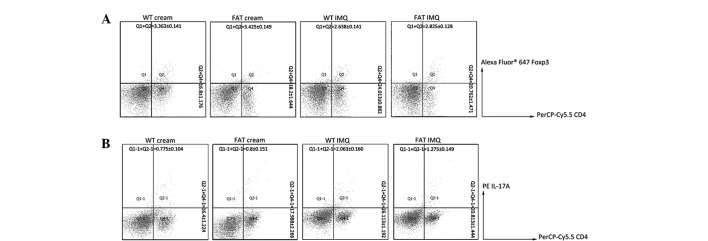
Changes in cellular composition of the mice spleen induced by IMQ treatment. Data from flow cytometry were analyzed for the percentage of CD4^+^T cells, Th17 cells and Treg cells. (A) Percentage of positive Treg cells and (B) percentage of positive Th17 cells. Figures show the percentage of positive Th17 cells which is increased in IMQ-induced mice, Treg cells by contrary. IMQ, imiquimod; WT, wild-type; Th17, T helper 17 cells; Treg, regulatory T cells.

The percentage of CD4^+^T cells, IL-17A and Foxp3 of the spleens of the four groups of mice was detected by flow cytometry. IL-17A and Foxp3 were secreted by Th17 cells and Treg cells, respectively, and therefore represent the quantity of Th17 cells and Treg cells. An increase in the percentage of IL-17A in IMQ-treated groups was observed through the amount of IL-17A^+^ following IMQ treatment (Fig. 5), whereas the percentage of Foxp3 was decreased. Additionally, the percentage of Th17 cells in fat-1 IMQ group were significantly lower (P<0.05) in spleens than in the WT IMQ group and the percentage of Treg cells in the spleens of fat-1 IMQ mice was significantly higher (P<0.05) compared with that in WT IMQ mice. However, there was no significant difference (P>0.05) between WT and fat-1 control mice in the cellular composition of the spleen. These results imply that endogenous n-3 PUFAs could upregulate the Foxp3 levels and reduce IL-17A to inhibit the inflammatory response.

### Effect of endogenous n-3 PUFAs on IMQ-induced increased proliferation and altered differentiation of keratinocytes

**Figure 6:**
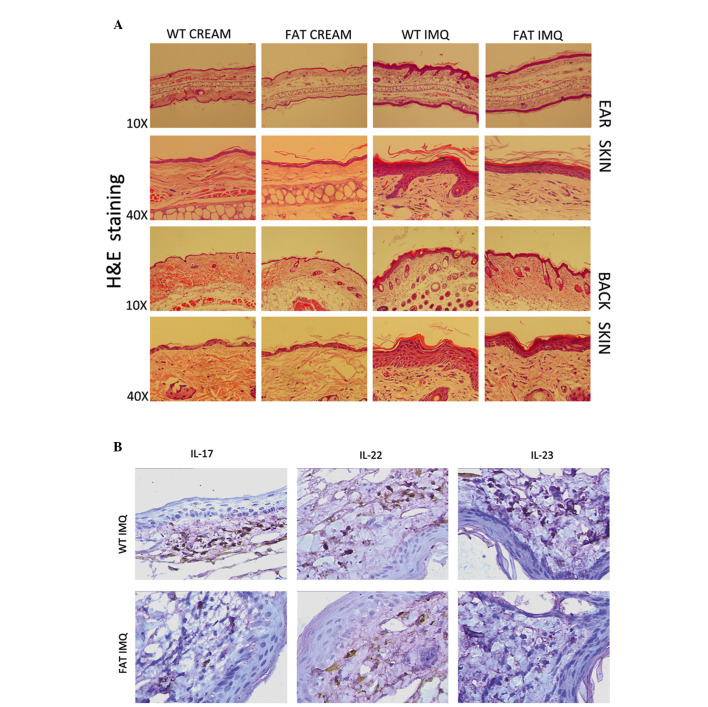
Topical IMQ alters keratinocyte differentiation and proliferation. Fat-1 and WT mice were treated for 14 consecutive days with IMQ or Vaseline.(A) Histological view (H&E staining and the higher magnification) of the ear and dorsal skin of the four groups of mice treated by two types of drugs. (B) Immunohistochemical analysis of inflammatory cytokines in the dorsal skin. IMQ, imiquimod; WT, wild-type; IL, interleukin.

It has been previously revealed that IMQ treatment induces increased epidermal thickening, hyperproliferous keratinocytes, parakeratosis and altered differentiated epidermis symptomatic of psoriatic skin lesions (19). In the present study, through the analysis of H&E-stained sections from the IMQ-treated dorsal tissue and ear skin, we observed increasing epidermal thickeness, stratum corneum, prickle cell layer in the IMQ-treated group compared with the control group (P<0.05) for the WT and fat-1 mice. However, the indication of inflammation of fat-1 mice (group D) was evidently weaker than in the WT mice (group B) in each respect (Fig. 6A). Stained-inflammatory factors secreted by Th17 cells from histological analyses confirmed multiplicity of IL-17, IL-22 and IL-23 accumulation in the WT IMQ-treated group vs. fat-1 IMQ-treated group (P<0.05; Fig. 6B). There was no evident change in groups A or C.

## Discussion

The IMQ-induced mouse is utilized as a model of human psoriatic lesions, as it exhibits similar characteristics, including erythema, epidermal thickening, scaling, neoangiogenesis, and the inflammatory infiltrate of T cells, neutrophils and dendritic cells (DCs). A previous study revealed that IMQ-induced skin inflammation markedly and consistently reflected the characteristics of psoriasis, including activated T cells, epidermal alterations by keratinocyte hyperproliferation and differentiation, existence of inflammatory cells consisting of T cells, neutrophils, DCs and vascular proliferation (17).

A study by van der Fits *et al* provided new insights into how Th1-Th17 challenge and IL-17 receptor signaling are critical to the development of psoriasis, since genetic knockout of these molecules leads to nearly a complete blockade of disease. Another study revealed that blockade of phosphatidylinositol 3-kinase (PI3K)δ or PI3Kγ ameliorated IMQ-induced psoriasis-like dermatitis, correlating with reduced IL-17 levels in the spleen serum and lesions (20). To further investigate the effect of IL-17 signaling in psoriasis, El Malki *et al* generated IL-17 receptorA deficient IL-17RA (del) mice and treated these mice with IMQ (4). The authors identified that psoriatic skin was partly reduced and delayed when compared with the controls. Of note, in the naive state, the skin of IL-17RA (del) mice contained markedly elevated numbers of Th17- and IL-17-producing γδ T cells. It is assumed that IL-17RA signaling regulates the population size of Th17 and γδ T cells. Therefore, the IL-23/IL-17 axis is critical in psoriasis-like lesions, which are triggered by the interaction between immune mediators from innate mechanisms and adaptive immunity. Therefore, in current studies, this classical IMQ-induced psoriasis model of mice is selected to examine the beneficial effects of n-3 PUFAs in psoriasis.

It has been reported that populations which consume a diet high in fish and other marine-based products, have a lower risk of heart disease. As a result, n-3 fatty acids as nutritional supplements have attracted notable attention and numerous studies have since focused on deciphering the beneficial effects of n-3 fatty acids in a number of different disease conditions (18,21). The relative ratio between n-6 and n-3 FAs is important in the overall health benefits of consuming n-3 FAs. The n-6 FAs, particularly arachidonic acid (AA), is a precursor of leukotrienes, prostaglandins (PGs) and other related compounds, affecting the synthesis of eicosanoids, which may enhance inflammation (19). In our previous study, it was identified that elongation and desaturation were inhibited by the presence of n-3 FA because the reduced levels of AA in cell membranes in fat-1 mice were partially replaced by EPA and DHA due to consuming increased amounts of n-3 PUFAs (19,22). This results in decreased production of pro-inflammatory mediators by AA, including PGE2. Therefore, n-3 PUFAs may restrain the hyperkeratosis and parakeratosis in psoriasis via reducing the levels of circulating inflammatory mediators. We also previously demonstrated that dietary n-3 PUFAs fed mice exhibit less IMQ-induced Th17 cell changes accompanied by psoriasis-like lesions (20), however, the affect of endogenous n-3 PUFAs on the psoriatic lesions remains poorly understood. To improve our understanding of the effect of endogenous n-3 PUFAs, a transgenic fat-1 gene overexpression mice model was used, which exhibit a characteristic reduction in the n-6 to n-3 ratio of 1:1, as compared with WT mice littermates with a ratio of 20–30:1, which may be optimal for health. Therefore, for the first time, to the best of our knowledge, we introduce this model to investigate the pathogenesis underlying psoriasis, with its advantages of steady endogenous n-3 PUFA ratio.

In the present study, the effect of endogenous n-3 PUFAs on psoriasis-like lesions was investigated in a fat-1 transgenic IMQ-induced model. The accuracy of grouping was guaranteed with PCR genotyping of the mice. The right ear and dorsal skin of the four groups of mice were separately treated with equal IMQ and cream for 14 consecutive days. As it may have been expected, the skin coated with IMQ exhibited thickening, erythema and scales, in the fat-1 transgenic mice, however, these inflammatory effects were more mild than those in WT mice. We noted that IMQ treatment resulted in hyperproliferative keratinocytes, parakeratosis, incrassate stratum corneum and stratum spinosum, all of which correspond with the characteristic histological features of psoriasis. The symptoms of the condition in fat-1 mice was notably weaker compared with the WT mice. Following this, we determined the anti-inflammatory effect of endogenous n-3 PUFAs and lower n-6/n-3 FA level for psoriasis-like lesions in fat-1 mice.

As the largest immune organ in body, the spleen participates in the process of systemic immune adjustment. In measuring the spleens, it was identified that those in the IMQ-treated mice were approximately twice the size and weight of those in the control groups, which provided evidence that inflammation increases the size, weight and cell number of the spleen. The evident changes observed in the spleens of fat-1 IMQ-treated mice may be due to endogenous n-3 PUFAs stimulating a more aggressive and active immune response. With a balanced role in the majority of the inflammatory reaction, Treg cells are usually presented by anti-inflammatory factor Foxp3. Under the condition of IMQ-treatment, the amount of Treg cells reduced, but the Th17 cells exhibited an opposing response. Fat-1 and wild-type mice had similar T cell and cytokine levels *in vivo*, as well as the presence of keratinocytes in the skin. We observed that the mildly decreased levels of Foxp3 secreted by regulatory T cells in the spleen of fat-1 IMQ-treated mice was accompanied by a lower level IL-17A from Th17 cells than that of the WT IMQ-treated group. It is believed that higher levels of n-3 PUFAs and lower n-6/n-3 ratios could maintain regulatory T cells and reduce the increasing Th17 cells in the spread of inflammation when compared with that of WT mice. Th17 cells are an important source of inflammatory factors, including IL-17, IL-22 and IL-23, that may all have an effect on psoriasis, which leads to hyperkeratosis and parakeratosis. Th17 cells not only specifically secrete IL-17, IL-22 and IL-23 but also are involved in promoting the differentiation from CD4^+^T cells. With regard to the affect of fatty acid on psoriasis, the levels of inflammatory factors in serum should always be considered. In the blood serum, IL-17, IL-22 and IL-23 levels were enhanced in the IMQ-treated mice, as compared with the control groups. In the fat-1 IMQ-induced group, the factors remained at lower levels compared with those of the WT IMQ-induced mice. It may be due to the impact of endogenous n-3 PUFAs on regulatory T cells and Th17 cells. In the present study, a significantly lower expression of IL-17, IL-22 and IL-23 was observed in the dorsal and ear skin of IMQ-treated fat-1 mice when compared with the WT mice, which confirmed the inhibitory impact of endogenous n-3 PUFAs on epidermis inflammation theoretically.

The results from the present study have revealed that endogenous lowering of the n-6/n-3 ratio and higher n-3 PUFA levels not only suppresses Th17 cells and maintains the level of anti-inflammatory cytokines Foxp3 from Treg cells, but also inhibits the expression of pro-inflammatory or inflammatory cytokines, including IL-17, IL-22 and IL-23 in the serum, preventing their accumulation in the lesion, and subsequently reducing thickening, erythema and scales. These data indicate the potential beneficial effects of endogenous n-3 PUFAs on IMQ-induced psoriasis. Currently the anti-psoriasis drugs, such as infliximab, have been widely used in prevention and treatment of TNF-α targeted prevention and treatment of psoriasis. One study identified that dietary n-3 PUFAs induced moderate clinical improvement and inhibited the inflammation in psoriasis (23). In the present study, the fat-1 transgenic mouse was selected to expound the molecular mechanisms underlying n-3 PUFA effects in psoriasis, due to its advantages over other models, in eliminating confounding factors with regard to exogenous diets. This study on fat-1 transgenic mice provided compelling evidence that the IL-17/IL-23 axis is a critical therapeutic target of inflammation in psoriasis and endogenous n-3 PUFAs are potential candidates for the prevention of hyperkeratosis and parakeratosis.

Recently, fish oils rich in n-3 FAs have been approved by the FDA as a prescription drug to treat cardiovascular diseases and high triglyceride levels owing to its cardioprotective effect (24), anti-carcinogenic effect (25), triglyceride lowering effect (26) and protective effect against inflammatory diseases (16,27), as a supplementation. Future studies should investigate the effect of endogenous n-3 PUFAs on a genetic level.

## Abbreviations

ALA: alpha-linolenic acid
DCs: dendritic cells
DHA: docosahexaenoic acid
EPA: eicosapentaenoic acid
FDA: Food and Drug Administration
GC: gas chromatography
IMQ: imiquimod
IL: interleukin
n-3 FAs: n-3 fatty acids
PASI: Psoriasis Area and Severity Index
WT: wild-type
